# Parent-of-origin effects in *SOX2* anophthalmia syndrome

**Published:** 2011-11-24

**Authors:** Robert J. Osborne, Jennifer J. Kurinczuk, Nicola K. Ragge

**Affiliations:** 1 Population Genetics Technologies, Babraham Research Campus, Babraham, Cambridgeshire, UK; 2 National Perinatal Epidemiology Unit, University of Oxford, Old Road Campus, Headington, Oxford, UK; 3 Department of Biological and Medical Sciences, School of Life Sciences, Oxford Brookes University, Gipsy Lane Campus, Oxford, UK; Wessex Clinical Genetics Service, The Princess Anne Hospital, Southampton, and Faculty of Medicine, University of Southampton, Southampton, UK

## Abstract

**Purpose:**

Sex determining region Y (SRY)-box 2 (*SOX2*) anophthalmia syndrome is an autosomal dominant disorder manifesting as severe developmental eye malformations associated with brain, esophageal, genital, and kidney abnormalities. The syndrome is usually caused by de novo mutations or deletions in the transcription factor *SOX2*. To investigate any potential parental susceptibility factors, we set out to determine the parent of origin of the mutations or deletions, and following this, to determine if birth order or parental age were significant factors, as well as whether mutation susceptibility was related to any sequence variants in cis with the mutant allele.

**Methods:**

We analyzed 23 cases of de novo disease to determine the parental origin of *SOX2* mutations and deletions using informative single nucleotide polymorphisms and a molecular haplotyping approach. We examined parental ages for *SOX2* mutation and deletion cases, compared these with the general population, and adjusted for birth order.

**Results:**

Although the majority of subjects had mutations or deletions that arose in the paternal germline (5/7 mutation and 5/8 deletion cases), there was no significant paternal bias for new mutations (binomial test, p=0.16) or deletions (binomial test, p=0.22). For both mutation and deletion cases, there was no significant association between any single nucleotide polymorphism allele and the mutant chromosome (p>0.05). Parents of the subjects with mutations were on average older at the birth of the affected child than the general population by 3.8 years (p=0.05) for mothers and 3.3 years (p=0.66) for fathers. Parents of the subjects with deletions were on average younger than the general population by 3.0 years (p=0.17) for mothers and 2.1 years (p=0.19) for fathers. Combining these data, the difference in pattern of parental age between the subjects with deletions and mutations was evident, with a difference of 6.5 years for mothers (p=0.05) and 5.0 years for fathers (p=0.22), with the mothers and fathers of subjects with mutations being older than the mothers and fathers of subjects with deletions. We observed that 14 of the 23 (61%) affected children were the first-born child to their mother, with 10/15 of the mutation cases (66%) and 4/8 deletion cases (50%) being first born. This is in comparison to 35% of births with isolated congenital anomalies overall who are first born (p=0.008).

**Conclusions:**

Sporadic *SOX2* mutations and deletions arose in both the male and female germlines. In keeping with several genetic disorders, we found that *SOX2* mutations were associated with older parental age and the difference was statistically significant for mothers (p=0.05), whereas, although not statistically significant, SOX2 deletion cases had younger parents. With the current sample size, there was no evidence that sequence variants in cis surrounding *SOX2* confer susceptibility to either mutations or deletions.

## Introduction

Developmental eye malformations, including anophthalmia (clinical absence of the eye) and microphthalmia (small eye), are a major cause of visual impairment worldwide. These conditions are clinically heterogeneous, and can manifest as either purely ocular defects, or for more than half of cases, in association with systemic anomalies [1]. Frequently, the cases display non-Mendelian inheritance patterns, reflecting the likely importance of genetic background and environmental influences. The first causative genes, mostly transcription factors that control eye morphogenetic pathways, are beginning to be identified, with dominant, recessive, X-linked, and oligogenic mechanisms represented [2–14]. Evidence from knockout gene experiments in mice (JAX), cytogenetic events associated with eye anomalies, and the number of human syndromes that include anophthalmia or microphthalmia as a clinical feature (Oxford Dysmorphology Database 2.1), suggest that at least 200 human anophthalmia-microphthalmia (AM) syndromes may eventually be defined. Correlations between AM and parental exposure to environmental factors around the time of conception or early pregnancy have been postulated [15]. However, epidemiological studies, by their nature, group all AM conditions together regardless of cause (see for example [16–19]), whereas each might represent a distinct genetic pathway with its own set of parameters and risk factors. Identification of the molecular basis of individual syndromes provides an opportunity to explore how different mutational events arise. The first step is to determine parental origin as this is a prerequisite to understanding periconception or gestational risk factors that contribute to disease.

Herein we have set out to determine the parent of origin for one of the earliest anophthalmic disorders to be genetically defined, sex determining region Y (SRY)-box 2 (*SOX2*; OMIM 184429) anophthalmia syndrome. This condition is characterized by severe, often bilateral, ocular malformations including anophthalmia or microphthalmia with coloboma or cyst, optic nerve hypoplasia, and rarely, retinal dystrophy [5,6,10,20]. Clinical features also include several extraocular features, including poor growth, cognitive deficit, motor disorder, seizures, sensorineural hearing loss, mesial temporal brain malformations, anterior pituitary hypoplasia, horseshoe kidney, and male (and possibly female) genital abnormalities [5,6,10,20–22]. The syndrome is usually caused by haploinsufficiency of *SOX2*, a transcription factor with a key regulatory role in lens development [23,24]. Four families have been described where SOX2 mutations were inherited from a gonosomal mosaic mother [25–28]. All remaining reported cases of *SOX2* mutations and deletions have occurred de novo. However, there is no information about the parental origin of the mutation or deletion for any of these cases. We used haplotype analysis to determine the parent of origin of *SOX2* mutation and deletion cases, and to investigate their relationship with parental age and birth order. We also sought to determine whether any sequence variants adjacent to the SOX2 gene were associated with a susceptibility to mutation or deletion.

## Methods

### Cases

Informed consent for genetic and phenotypic analysis was obtained from the patient and parents, in accordance with approval by the Cambridgeshire 1 Research Ethics Committee 04/Q0104/129. Paternity was confirmed using the PowerPlex® 16 System (Promega, Southampton, UK). Cases 1–8 with *SOX2* deletions and cases 9–19 with *SOX2* mutations have been previously described [5,6,10]. The location of mutations and deletions are shown in Table 1. *SOX2* deletion and mutation cases were identified as de novo following analysis of peripheral blood samples; however, it is not possible to exclude parental gonosomal mosaicism. Case 19 has bilateral anophthalmia with micropenis, agenesis of the corpus callosum, and severe developmental delay, and is most likely to be the same case as previously reported [22]. The proband has a de novo heterozygous c.479delA (p.Tyr160Serfs*4) mutation. Cases 20–23 are previously unreported de novo *SOX2* mutations, as determined by analysis of peripheral blood. Case 20 is a male with right microanterior segment, sclerocornea, thin lens, posterior staphyloma with hand movement vision, and left extreme microphthalmia (axial length 10 mm) with congenital aphakia, short stature (normal pituitary function), low weight, unusual gait, muscle weakness, small fifth finger, dysmorphic features, slightly prominent nose, and has experienced fainting fits. He had a c.582_592dup (p.His198Argfs*9) mutation. Case 21 is a boy with isolated bilateral anophthalmia. There were no other systemic features apart from a slightly high arched palate. The proband has a c.143_144delinsAA mutation (p.Phe48*). Case 22 is a girl with isolated bilateral anophthalmia. She has a de novo c.70_89del (p.Asn24Argfs*65) mutation. Case 23 is a boy born at term with a normal birthweight, bilateral severe microphthalmia associated with funnel retinal detachments, and delayed motor development. He has a de novo c.542delC mutation (p.Pro181Argfs*22).

**Table 1:** Summary of cases and gene change.

**Case**	***SOX2* gene change**	**Reference**
1	Partial gene deletion	[10]
2	Whole gene deletion	[10]
3	Whole gene deletion	[10]
4	Whole gene deletion	[10]
5	Whole gene deletion	[10]
6	Whole gene deletion	[10]
7	Whole gene deletion	[10]
8	Whole gene deletion	[10]
9	c. 529C>T; p.Q177X	[5,6]
10	c.53 C>A mutation, p.S18X	[10]
11	c. 248C>A, p.S83X	[5,6]
12	c.70del20, p.N24fs88X	[10]
13	c.480C>G, p.Y160X	[10]
14	c.285dupG, p.K95fs109X	[10]
15	c. 529C>T; p.Q177X	[5,6]
16	c.290T>C; p.L97P	[6]
17	c.70del20 p.N24fs88X	[10]
18	c.188delA p.N63fs101X	[10]
19	c.479delA, p.S159fs163X	This study; same case likely published [22]
20	c.582–592dup, (p.His198Argfs*9)	This study
21	c.143–144TC>AA, (p.Phe48*)	This study
22	c.70del20 p.N24fs88X	This study
23	c.542delC, (p.Pro181Argfs*22)	This study

### Determining parent of origin

#### Single nucleotide polymorphism genotyping

Single nucleotide polymorphisms (SNPs) surrounding the *SOX2* genomic locale were genotyped to determine the parental origin of sporadic *SOX2* mutations and partial or whole gene deletions. To trace the parental origin of each *SOX2* deletion, we identified cases where affected subjects failed to inherit an SNP allele from one parent. We genotyped 24 SNPs within 50 kb of the *SOX2* gene in subjects 1–8 and their parents. A molecular haplotyping approach (described below) was used to determine the parent of origin for the SOX2 mutations.

Genomic DNA was prepared from peripheral blood samples using a QIAamp DNA Blood Mini kit (Qiagen, Crawley, UK). For mutation and deletion cases, we genotyped the following SNPs: rs13070015, rs9290727, rs4434184, rs6806029, rs12496378, rs12487748, rs12497248, rs35788479, rs35095647, rs11915160, rs4575941, and rs4459940 (SNP). For deletion cases, we also genotyped SNPs: rs13074951, rs13097472, rs1558797, rs1558798, rs34961466, rs36062376, rs4855037, rs6443761, rs6443762, rs6765739, rs7610679, and rs7633815. PCR products were generated for SNPs using the primers detailed in Table 2. PCR amplification was performed in a 50 µl reaction volume containing 100 ng DNA, 0.1 µM each primer, 5 µl 10× reaction buffer, 2 µl 25 mM MgCl_2_, 0.2 mM dNTPs, and 2.5 Units HotStarTaq DNA polymerase (Qiagen). Cycling parameters were 94 °C for 15 min followed by 35 cycles at 94 °C for 30 s, 60 °C for 30 s, 72 °C for 1 min, and a final extension at 72 °C for 10 min. PCR products were treated with ExoSapIT (USB) and sequenced using standard techniques.

**Table 2:** PCR primers for SNP analysis.

**SNP**	**Forward 5′-3′**	**Reverse 5′-3′**
rs13070015	CCAGGATTGGAGACCTTACTG	GGTTGGGGGAGAGGAGATAA
rs9290727	CAGCGTCTTGAATGCCCTCAG	GGATTCCTGGTCAACGAAATGC
rs4434184	ATCACTCAGACGGGCAGATAAG	TCAGATGGAGTTGGGTTTCC
rs6806029/rs12496378	TGCCAAACACATCAGCCCTCTG	GGGCTACCCTTTCACTGCAAGG
rs12487748/ rs12497248	AGCTGTAGTGCAGGGATAGAATCTTAAC	AGAACAGCTTTGCATTCAGCTCC
rs35788479	CGTGGAAGGGAAGTGTTACACTCC	GCCCACTCTATTTCAATCCAGCAC
rs35095647	AGGTAAGAGAGGAGAGCGGAAGAGC	CAGCAACAGGTCACACCACACG
rs11915160	GTACGGTAGGAGCTTTGCAGGAAG	TGTCCTAAATTTCAGCTGCAGAATC
rs4575941/rs4459940	GGGTCAAAGAGCAATGCTTCAA	CCCCTTTTGCCCCATATTGC
rs34961466/rs13074951/rs13097472	CCAAGCCTGTAGCCCCAAAT	GGTGGGTGGTGATGCAGAAG
rs4855037/rs1558797/rs1558798	GCTTGCAGTGAGCAGAGATCGC	TGGGACGCAAACCTTGACAGTC
rs7633815/rs36062376	GGAGTTTCTCATTCCCTGCTC	CTCTGCTTCCTGGGTTCAAG
rs6443761/rs6443762	GCCTTCACTCTGTCACCCTATCTG	TTTGGCCGGTTCTGAATCTGACTAC
rs6765739	AGGCTCGTGGCTTAGGAGATTG	AAATAGCCACTGAAAGGCAAGGTC
rs7610679	TCAAGAAGACAGAACTGCCAAGAGG	CCCTTCTTCTACCCTCCCATTCC
SOX2 5′ outer	TACAACATGATGGAGACGGAGCTGA	TGCGAGCTGGTCATGGAGTTG
SOX2 5′ inner	GGTAGCCCAGCTGGTCCTG	
SOX2 3′ outer	GGCGTGAACCAGCGCATGG	TCATTTGCTGTGGGTGATGGG
SOX2 3′ inner	GAGCGTACCGGGTTTTCTC	
rs11915160 outer	CCAATCCCATCCACACTCACG	TGTCCTAAATTTCAGCTGCAGAATC
rs11915160 inner	GTACGGTAGGAGCTTTGCAGGAAG	
rs4575941/rs4459940 outer	GGGTCAAAGAGCAATGCTTCAA	GGCAAAGGTTGTAAATGAGCACC
rs4575941/rs4459940 inner	CCCCTTTTGCCCCATATTGC	

#### Molecular haplotyping

We used a molecular haplotyping approach [29] to determine the parental origin of each *SOX2* mutation. We genotyped 12 SNPs within 20 kb of the *SOX2* gene in the *SOX2* mutation subjects and their parents, and identified at least one informative SNP for seven subjects. For each of the seven informative subjects, genomic DNA at limiting dilution was used to generate a panel of 45 aliquots (and one negative control). Each aliquot was amplified using primers for both the *SOX2* CoDing Sequence (CDS) and an informative SNP locus (rs11915160, rs4459940, or rs4575941) in a single multiplex PCR. PCR products were then diluted and the *SOX2* CDS and SNP locus were amplified in separate monoplex PCR reactions using heminested primers. For DNA samples, 47/94±6.19 (standard deviation [SD])] aliquots were positive for the *SOX2* CDS or the informative SNP locus. Assuming a Poisson distribution of DNA fragments among the samples [29], this implies a mean of 0.71±0.14 (SD) autosomal genomes per aliquot. Aliquots positive for the presence of both the *SOX2* CDS and the informative SNP locus were then sequenced to determine which SNP variant was associated with the mutant *SOX2* allele. A small number of aliquots were derived from both haplotypes because they were heterozygous for either the *SOX2* mutation or the SNP locus; these did not contribute to determine parental origin. Individual haplotypes were reconstructed by inspection of the data and the parental origin determined in each case. For each subject, the haplotype was supported by odds of at least 10^9^:1.

The multiplex first-phase PCR contained primers for *SOX2* and one informative SNP (rs11915160, rs4459940, or rs4575941). Alternative *SOX2* primers were used depending on the position of the mutation. The first-phase PCR was performed in a 10 µl reaction containing 1 µl DNA (at one genome equivalent [3 pg/µl]), 0.04 µM each external-forward and -reverse primer, 10X reaction buffer, 1 mM MgCl_2_, 0.2 mM dNTPs and 0.5 Units HotStarTaq DNA polymerase (Qiagen). Cycling conditions were 94 °C for 15 min followed by 35 cycles at 94 °C for 30 s, 60 °C for 30 s, 72 °C for 1 min, and a final extension at 72 °C for 10 min. First-phase reaction products were diluted to 150 µl with water and 3 µl aliquots were used as template in second-phase PCR reactions to independently assay *SOX2* and each SNP. Second-phase PCR was performed in a 20 µl reaction containing template DNA, 0.2 µM each internal-forward and external-reverse primer, 2 µl 10X reaction buffer, 1 mM MgCl_2_, 0.2 mM dNTPs, and 1 Unit HotStarTaq DNA polymerase (Qiagen). Cycling conditions were the same as for the first-phase PCR. Products were analyzed by gel electrophoresis for the presence/absence of the expected PCR product. PCR products from aliquots positive for *SOX2* and the SNP locus were treated with ExoSapIT (USB, High Wycombe, UK) and sequenced using standard techniques [11,12].

The confidence that can be assigned to the linkage phase between the *SOX2* and SNP loci was estimated using calculations described by Konfortov et al. [29]. This measure depends on the physical distance between two loci, the average fragment size of DNA in the aliquots, the number of autosomal genomes per aliquot, and the number of genotyping results that agree or conflict with a given haplotype phase. The physical distance between each *SOX2* mutation and the SNP loci was calculated for each assay using genomic positions from the UCSC genome browser [30]. Template DNA was prepared using the QIAamp DNA Blood Mini kit (Qiagen), which predominantly yields fragments of 20–30 kb; therefore, we conservatively estimated average DNA fragment size at 20 kb. The number of autosomal genomes per aliquot was estimated for each sample from the total number of positive aliquots in the second-phase PCR using the Poisson distribution [29].

### Sequence variant analysis

To examine whether particular sequence variants predispose individuals to the occurrence of mutations or deletions, we used parental data to determine the genotype of SNPs located on mutant (mutated/deleted) and wild-type (nonmutated/nondeleted) chromosomes. For deletion cases, we analyzed 24 SNPs, and for mutation cases 12 SNPs, previously genotyped for parental origin. We compared the frequency of alleles on mutant versus wild-type chromosomes using Fisher’s exact test.

### Parental age

To examine parental age effects in *SOX2* anophthalmia syndrome, the median ages of parents of affected subjects were compared with median parental age at birth of the general population using the national birth cohorts of England and Wales for the relevant years of birth of the affected subjects; paternal age was not available for two of the children. Given the sparse numbers in the affected group, the distribution of parental age is uncertain and therefore medians were calculated and compared using the Mann–Whitney U-test (Table 3). General population comparison data were obtained from the national birth statistics for England and Wales published by the Office for National Statistics, Birth Statistics 1985 to 2007, Series FM1, London: ONS, 1988 to 2009.

**Table 3:** Parental age at the birth of the anophthalmia subjects compared with parental age in the general population of births for England and Wales.

**Parental age comparisons**	**Number of anophthalmia subjects**	**Parents of anophthalmia subjects**	**Parental age at birth for the general population^3^**	**Difference in age (years)**	**p value^4^**
**Deletions**					
Median (range) paternal age in years	8	30.0 (24.0–34.0)	32.1 (30.0–32.2)	−2.1^1^	0.19
Median (range) maternal age in years	8	24.5 (21.0–30.0)	27.5 (26.5–31.0)	−3.0^1^	0.17
**Mutations**					
Median (range) paternal age in years	13	35.0 (18.0–44.0)	31.7 (29.7–32.4)	+3.3^1^	0.66
Median (range) maternal age in years	15	31.0 (18.0–41.0)	27.2 (25.1–32.4)	+3.8^1^	0.05
**Paternal age**					
Median (range) paternal age in years of subjects with deletions	8	30.0 (24.0–34.0)	-	+5.0^2^	0.22
Median (range) paternal age in years of subjects with mutation	13	35.0 (18.0–44.0)	-		
**Maternal age**					
Median (range) maternal age in years of subjects with deletions	8	24.5 (21.0–30.0)	-	+6.5^2^	0.05
Median (range) maternal age in years of subjects with mutation	15	31.0 (18.0–41.0)	-		

^1^The difference is the median parental age of anophthalmia subjects minus the median parental age in the general population. ^2^The difference is the median parental age of anophthalmia mutation cases minus the median parental age of anophthalmia deletion cases. ^3^Median of the birth year-specific general population means. ^4^P-values derived from the Mann–Whitney U test.

Fourteen of the 23 children were first births to their mothers. To take into account the potential confounding effect of birth order, the birth order–specific mean maternal age of the general population was extracted for the birth year of each affected child. Data to enable birth order to be accounted for in relation to paternal age are not published; thus, the overall mean general population paternal age was extracted for the year of birth of each of the affected children. Data on birth order of the affected children were compared with data from the Congenital Anomalies Register for Oxfordshire, Berkshire and Buckinghamshire (CAROBB), as cases from the England and Wales National Congenital Anomalies System are known to be underascertained and not published by birth order.

## Results

### Parent of origin

Deletion cases: The majority of subjects (5/8) had deletions that arose in the paternal germline, but this did not represent a statistically significant paternal bias to the a priori assumption that deletions occur with the same probability on maternal and paternal chromosomes (binomial test, p=0.22).

Mutation cases: In the majority of subjects (5/7), the mutation was paternal in origin, although this does not represent a statistically significant paternal bias to the a priori assumption that mutations occur with the same probability on maternal and paternal chromosomes (binomial test, p=0.16).

For both mutation and deletion cases, there was no significant association between any SNP allele and the mutant chromosome (p>0.05). With the current sample size, there is no evidence that *in cis* sequence variants surrounding *SOX2* confer susceptibility to either mutations or deletions.

We were unable to determine parental origin in the remaining eight cases because SNPs were uninformative (4 cases) or DNA samples were unavailable (4 cases).

### Parental age

Parents of the subjects with mutations were on average older at the birth of the affected child than the general population by 3.8 years (p=0.05) for mothers and 3.3 years (p=0.66) for fathers. In contrast, the parents of the subjects with deletions were on average younger than the general population by 3.0 years (p=0.17) for mothers and 2.1 years (p=0.19) for fathers. The difference in pattern of parental age was evident in the comparison of parental age between the subjects with deletions and mutations, with a difference of 6.5 years for mothers (p=0.05) and 5.0 years for fathers (p=0.22), with the mothers and fathers of subjects with mutations being older than the mothers and fathers of subjects with deletions. We observed that 14 of the 23 (61%) affected children were the first-born child to their mother, with 10/15 of the mutation cases (66%) and 4/8 deletion cases (50%) being first born. This is in comparison to 35% of births with isolated congenital anomalies overall who are first born (p=0.008; P Boyd, CAROBB, personal communication).

## Discussion

The parental origin of de novo genetic anomalies depends on several factors. First, the type of genetic defect is often parent specific. Spontaneous base substitutions are thought to occur preferentially in the paternal, rather than the maternal, germline because of the higher number of cell divisions in spermatogenesis than oogenesis [31]. This is supported by evidence from several disorders, where spontaneous base substitutions arise predominantly in the paternal germline [32,33]. In contrast, certain spontaneous chromosomal abnormalities are more commonly maternal in origin, such as those causing Duchenne muscular dystrophy or neurofibromatosis [34,35]. Second, a genetic anomaly might have a selective advantage or disadvantage in male or female germ cells. For example, positive selection of sperm with mutations in *FGFR2* in the male germline is thought to contribute to the paternal age effect in Apert syndrome [36]. Third, parental origin might depend on which parent has been exposed to environmental hazards that cause germline mutations [37].

*SOX2* anophthalmia syndrome can occur through structural rearrangement of 3q21 [5,38,39], whole or partial gene deletions [10], intragenic insertions, deletions, or point mutations [5,6,10,20–22,39,40], leading to SOX2 haploinsufficiency. The majority of cases are de novo, with occasional cases resulting from parental gonosomal mosaicism [25–28]. A potential parental bias for gonosomal mosaicism exists because all cases reported to date are maternal in origin. Nevertheless, it is still necessary to test both parents of a proband, preferably using more than one tissue type (blood and buccal cells) to determine if a parent is mosaic to enable accurate genetic counselling. There have been no described cases of inherited *SOX2* mutations or deletions, most likely because of reduced genetic fitness, in addition to male and possibly female genital abnormalities, some related to hypothalamic-pituitary axis dysfunction.

The majority of the informative *SOX2* anophthalmia mutation (5/7) and deletion (5/8) cases were paternal in origin, although this does not represent a statistically significant bias in distribution in favor of paternal origin. Previous studies have identified disorders with an exclusively paternal origin, such as achondroplasia and Apert syndrome [41,42], in addition to those with weaker paternal origin effects, such as neurofibromatosis, retinoblastoma, Sotos syndrome, and Treacher-Collins syndrome [43–46]. Disorders with an exclusively paternal origin are characterized by a narrow mutational range. For example, >99% of sporadic achondroplasia is caused by p.Gly380Arg mutations in *FGFR3* [47,48] and >98% of sporadic Apert syndrome is caused by p.Ser252Trp or p.Pro253Arg mutations in *FGFR2* [41,49]. Typically, disorders with weaker paternal origin effects have wider mutational ranges, with base substitutions comprising a smaller proportion of the total number of mutations. For example, one study found that only 42% of retinoblastoma germline mutations are base substitutions, with 15% comprising large deletions and 26% small insertions/deletions [50].

Consistent with the absence of a significant paternal origin effect, *SOX2* anophthalmia syndrome is characterized by a wide mutation spectrum. Of the 15 cases examined here, there were 13 different mutations. Only seven mutations were base substitutions, with the remainder single nucleotide (three) or polynucleotide (three) additions or deletions. Furthermore, none of the mutation events in our cohort is a C>T transition at a CpG dinucleotide, which occur when methylated cytosine is deaminated to thymine. The absence of C>T transitions at CpG dinucleotides is unusual given that the *SOX2* coding sequence overlaps with a CpG island [51]. Only two identified mutations in our cohort were present in more than one individual: c.529C>T (p.Gln177*) and c.70_89del (p.Asn24Argfs*65) in published cases 12 and 17, and the new case 22. The c.70_89del (p.Asn24Argfs*65) mutation has also been noted in eight other published anophthalmia cases summarized by Schneider and colleagues [28], including one pair of monozygotic twins discordant for anophthalmia [40]. The similar c.70_86del (p.Asn24Glyfs*66) mutation has also been noted in two siblings discordant for anophthalmia [26]. The c.70_89del (p.Asn24Argfs*65) mutation results in the deletion of one of a pair of direct 5′-[GCG]_3_GC-3′ repeats, and the intervening nine nucleotides. The c.70_86del (p.Asn24Glyfs*66) mutation results in the deletion of one of the 5′-[GCG]_2_GC-3′ repeats, and the intervening nine nucleotides. We were able to determine parental origin for one of the c.70_89del (p.Asn24Argfs*65) cases in our cohort, and found that the mutation was paternally derived. Previous studies have found that this type of mutation is predominantly paternal in origin and arises during replication by a mispairing of direct repeats (see [52]).

Sporadic cases of several disorders are associated with older paternal age (reviewed in [32]) or rarely with younger maternal age [53–55]. We found the mutation cases were associated with older parental age, although this was only statistically significant for the mothers. The small number of mutation cases of paternal origin (n=13) means that chance cannot be excluded as an explanation for this finding, but the absence of a paternal age effect is consistent with the absence of a paternal origin bias. One possible explanation for the maternal age association is that DNA repair mechanisms are maternally derived from mRNA and protein in the oocyte. For example, female mice deficient in double-stranded break repair do not efficiently repair double-strand breaks that originally arose in sperm [56,57]. Therefore, the ability of the mother to correct a genetic defect at oogenesis or during the first cell division after zygosis is critical [58], and this may be impaired with increasing age.

In contrast, we found the deletion cases had parents who were on average younger by two to three years than parents in general, and this difference is substantial in population terms. These results were, however, not statistically significant; that is, with only eight cases in total, these comparisons lacked the statistical power to unmask what might be a real and important finding that merits further investigation. Younger maternal age has previously been noted in several disorders, including septo-optic dysplasia [53], cleft lip, central nervous system anomalies, gastroschisis, and skin, upper limb and female genital anomalies [54,59,60]. One possible reason for this is a greater likelihood of the survival of malformed conceptuses in younger women, which is consistent with the finding that the frequency of chromosomally normal spontaneous abortions increases with maternal age [61]. Several studies have also drawn attention to the role of lifestyle factors in the etiology of anomalies associated with younger maternal age. For example, drug and alcohol consumption and cigarette smoking have been implicated in the risk of specific congenital anomalies that tend to occur predominantly in younger women, for example, gastroschisis [60,62] and optic nerve hypoplasia [55,63,64]. With our relatively small number of cases, we were unable to explore further the relationship between lifestyle and/or environmental factors and the occurrence of *SOX2* deletions or mutations. Such investigations will require an international collaborative effort to identify sufficient cases to explore this further. However, this would clearly be worthwhile if this is able to shed light on the causal mechanisms leading to *SOX2* mutational events, which have such a profound impact on the children and families affected.
